# A new species of *Liphistius* Schiødte, 1849 (Araneae, Liphistiidae) from Yunnan, China

**DOI:** 10.3897/BDJ.11.e113290

**Published:** 2023-11-10

**Authors:** Yejie Lin, Shuqiang Li

**Affiliations:** 1 Hebei Key Laboratory of Animal Diversity, College of Life Science, Langfang Normal University, Langfang, Hebei 065000, China Hebei Key Laboratory of Animal Diversity, College of Life Science, Langfang Normal University Langfang, Hebei 065000 China; 2 Institute of Zoology, Chinese Academy of sciences, Beijing 100101, China Institute of Zoology, Chinese Academy of sciences Beijing 100101 China

**Keywords:** diagnosis, Asia, spider, type

## Abstract

**Background:**

The spider genus *Liphistius* Schiødte, 1849 contains 69 species, endemic to Indochina and Southeast Asia. Only one species is currently known from the Chinese province of Yunnan: *Liphistiusnabang* Yu, Zhang & Zhang, 2021.

**New information:**

A new species, *Liphistiusliz* Lin & Li, sp. nov., is described from Yunnan, China, on the basis of both sexes. Photos and a morphological description of the new species are provided.

## Introduction

Heptathelidae Kishida, 1923 and Liphistiidae Thorell, 1869 are the extant families of the suborder Mesothelae Pocock, 1892 in the Araneae Clerck, 1757 and the most basal lineage of all existing spiders (Li 2022). The Liphistiidae, with the latter containing the single genus *Liphistius* Schiødte, 1849, can be distinguished from Heptathelidae by the presence of clavate trichobothria on the leg tarsi and palpal tarsi, the male palp having a tibial apophysis and the female genitalia with a dorsal receptacular cluster on a ventral poreplate (Kraus 1978, Yu et al. 2021).

Chinese spider taxonomists have published a large number of papers in the 21^st^ century, but due to the rich biodiversity of the Chinese territory, there are still many unknown species (Li et al. 2021, Yao et al. 2021, Yang et al. 2021, Li 2022, Liu et al. 2022, Lu et al. 2022, Zhao et al. 2022). Presently, *Liphistius* comprises 69 species, endemic to Indochina and Southeast Asia (WSC 2023) and only one species, *L.nabang* Yu, Zhang & Zhang, 2021 (Yunnan), was reported from China. In this paper, we report the second species from Yunnan, China: *L.liz* sp. nov.

## Materials and methods

All specimens were preserved in 80% ethanol. The spermathecae were cleared in trypsin enzyme solution to dissolve non-chitinous tissues. Specimens were examined under a Leica M205C stereomicroscope. Photographs were taken with an Olympus C7070 zoom digital camera (7.1 megapixels). Photographs were stacked with Helicon Focus (v. 7.6.1) or Zerene Stacker (v. 1.04) and processed in Adobe Photoshop CC2022.

All measurements are in millimetres (mm) and were obtained with an Olympus SZX16 stereomicroscope with a Zongyuan CCD industrial camera. All measurements of body lengths do not include the chelicerae. Eye sizes are measured as the maximum diameter from either the dorsal or the frontal view. Legs were measured laterally. Leg measurements are given as follows: total length (femur, patella+tibia, metatarsus, tarsus). The terminology used in the text and figures follows Schwendinger (1995).

A total of 1533 bases of cytochrome oxidase I were sequenced by using the following primers: ExtA (5’-GAAGTTTATATTTTAATTTTACCTGG-3’) and ExtB (5’-CCTATTGA WARAACATARTGAAAATG-3’). This PCR profile consisted of an initial denaturing step at 94°C for 2 min, 30 amplification cycles [94°C for 30 s, 50°C or optimal annealing temperature (Tm°C) for 45 s, 72°C for 45 s], followed by a final extension step at 72°C for 5 min.

Types from the current study are deposited in the Institute of Zoology, Chinese Academy of Sciences in Beijing (**IZCAS)**.

Abbreviations used in text: **ALE**, anterior lateral eye; **AME**, anterior median eye; **PLE**, posterior lateral eye; **PME**, posterior median eye.

## Taxon treatments

### Liphistius liz

Lin & Li, 2023 sp. nov.

19E347F1-AAD2-59EB-BEEB-EAC4E2814081

91074358-F13D-418A-9890-8A25B4B73FC2

#### Materials

- **Type status:** Holotype. **Occurrence:** catalogNumber: IZCAS-Ar44748; recordedBy: Yicheng Lin; individualCount: 1; sex: male; lifeStage: adult; occurrenceID: 5BCC41FF-4DC2-53C5-836F-F9BBC80D4BDE; **Taxon:** scientificName: *Liphistiusliz*; **Location:** country: China; stateProvince: Yunnan; county: Lianghe; locality: Jiubao Achang Township, Shizunao; verbatimElevation: 1200 m; decimalLatitude: 24.7478; decimalLongitude: 98.2106; **Identification:** identifiedBy: Yejie Lin; dateIdentified: 2023; **Event:** year: 2023; month: 5; day: 13
- **Type status:** Paratype. **Occurrence:** catalogNumber: IZCAS-Ar44749; recordedBy: Yicheng Lin; individualCount: 1; sex: female; lifeStage: adult; occurrenceID: 2177DB32-CFCD-5FED-9AAF-D1629797C869; **Taxon:** scientificName: *Liphistiusliz*; **Location:** country: China; stateProvince: Yunnan; county: Lianghe; locality: Jiubao Achang Township, Shizunao; verbatimElevation: 1200 m; decimalLatitude: 24.7478; decimalLongitude: 98.2106; **Identification:** identifiedBy: Yejie Lin; dateIdentified: 2023; **Event:** year: 2023; month: 8; day: 12
- **Type status:** Paratype. **Occurrence:** catalogNumber: IZCAS-Ar44750; recordedBy: Yicheng Lin; individualCount: 1; sex: female; lifeStage: adult; occurrenceID: 4FEE7ED6-6BCF-50BB-A7A5-D3C318237341; **Taxon:** scientificName: *Liphistiusliz*; **Location:** country: China; stateProvince: Yunnan; county: Lianghe; locality: Jiubao Achang Township, Shizunao; verbatimElevation: 1200 m; decimalLatitude: 24.7478; decimalLongitude: 98.2106; **Identification:** identifiedBy: Yejie Lin; dateIdentified: 2023; **Event:** year: 2023; month: 8; day: 12
- **Type status:** Paratype. **Occurrence:** catalogNumber: IZCAS-Ar44751; recordedBy: Yicheng Lin; individualCount: 1; sex: female; lifeStage: adult; occurrenceID: 34FCBAD1-1985-59EA-8784-A3605859BC42; **Taxon:** scientificName: *Liphistiusliz*; **Location:** country: China; stateProvince: Yunnan; county: Lianghe; locality: Jiubao Achang Township, Shizunao; verbatimElevation: 1200 m; decimalLatitude: 24.7478; decimalLongitude: 98.2106; **Identification:** identifiedBy: Yejie Lin; dateIdentified: 2023; **Event:** year: 2023; month: 8; day: 12
- **Type status:** Paratype. **Occurrence:** catalogNumber: IZCAS-Ar44752; recordedBy: Yicheng Lin; individualCount: 1; sex: female; lifeStage: adult; occurrenceID: BB3338CB-0A61-516F-BEA2-1CA2A06BA8E9; **Taxon:** scientificName: *Liphistiusliz*; **Location:** country: China; stateProvince: Yunnan; county: Lianghe; locality: Jiubao Achang Township, Shizunao; verbatimElevation: 1200 m; decimalLatitude: 24.7478; decimalLongitude: 98.2106; **Identification:** identifiedBy: Yejie Lin; dateIdentified: 2023; **Event:** year: 2023; month: 8; day: 12

#### Description

Male (holotype, Figs 2, 3b, 4, 7A). Total length 7.55. Carapace 4.19 long and 3.83 wide, earthy yellow in ethanol (slightly lighter than in life), margin and fovea colour darker, without obvious dark stripes between coxal elevations (Fig. 7A). Eye sizes and interdistances: AME 0.06, ALE 0.49, PME 0.25, PLE 0.35, AME–AME 0.08, AME–ALE 0.08, PME–PME 0.04, PME–PLE 0.06, AME–PME 0.02, ALE–PLE 0.05. Chelicerae reduced, brown, with several short macrosetae. Labium 0.73 long and 0.44 wide, fused with sternum. Sternum 1.98 long and 0.75 wide, posterior tip elongated. Opisthosoma 3.54 long and 2.29 wide, with ten tergites. Leg measurements: leg I 11.86 (3.26, 3.85, 3.17, 1.58), leg II 13.46 (3.83, 4.07, 3.51, 2.05), leg III 14.88 (3.53, 4.30, 4.47, 2.58), leg IV 19.41 (4.69, 5.51, 5.91, 3.30).

Palp (Figs 2, 3b, 4). Tibial apophysis of palp almost as high as wide, situated near retrolateral margin of tibia, with four megaspines. Cymbium with two clavate trichobothria retrolaterally (Fig. 4D). Paracymbium large and thick, almost as wide as cymbium, cumulus distinctly elevated with many long setae (Fig. 4). Subtegulum curved in prolaterodorsal and ventral views, without obvious apophysis. Tegulum with a well-developed and denticulate distal edge. Half of the contrategulum strongly sclerotised, with a ventral process (Figs 2, 3b). Paraembolic plate slightly elevated. Embolus partly sclerotised, with some longitudinal ridges extending to the tip, margins of these ridges slightly dentated (Figs 2, 3b).

**Figure 1.**
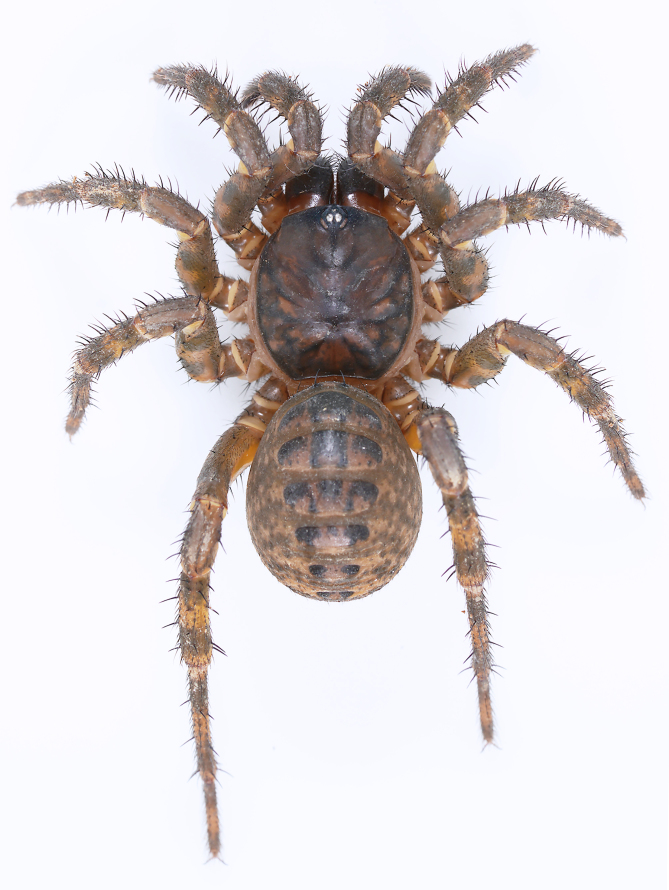
*Liphistiusliz* sp. nov., paratype female, in life.

Female (paratype, Figs 1, 5, 7B). Total length 10.32. Carapace 4.87 long, 4.16 wide, colour as in males, except shades being darker (Figs 1, 7B). Eye sizes and interdistances: AME 0.06, ALE 0.45, PME 0.27, PLE 0.31, AME–AME 0.06, AME–ALE 0.07, PME–PME 0.04, PME–PLE 0.05, AME–PME 0.04, ALE–PLE 0.05. Chelicerae robust, reddish-brown, with a few short stripes on dorsal side and several long macrosetae on retrolateral edge of fang groove. Labium 1.03 long, 0.52 wide. Sternum 242 long, 1.23 wide. Opisthosoma 5.92 long, 4.52 wide, with ten tergites. Leg measurements: leg I 8.60 (3.04, 2.77, 1.75, 1.04), leg II 8.63 (2.68, 3.16, 1.65, 1.14), leg III 9.80 (2.98, 3.14, 2.28, 1.48), leg IV 14.34 (3.93, 4.47, 3.83, 2.11).

Vulva (Fig. 5): Poreplate with four notobvious protuberances (two anterolateral and two posterolateral), two posterolateral protuberances not attached to ventral rim of poreplate. Central dorsal opening globular, receptacular cluster grape-shaped. Bulging margins on ventral poreplate only extending to the posterolateral corner of poreplate (Fig. 5B) and distance between bulging margins almost as wide as poreplate. Genital atrium straight. Posterior area of posterior stalk located in the same plane of poreplate and almost as wide as poreplate (Fig. 5A).

#### Diagnosis

**Figure 2.**
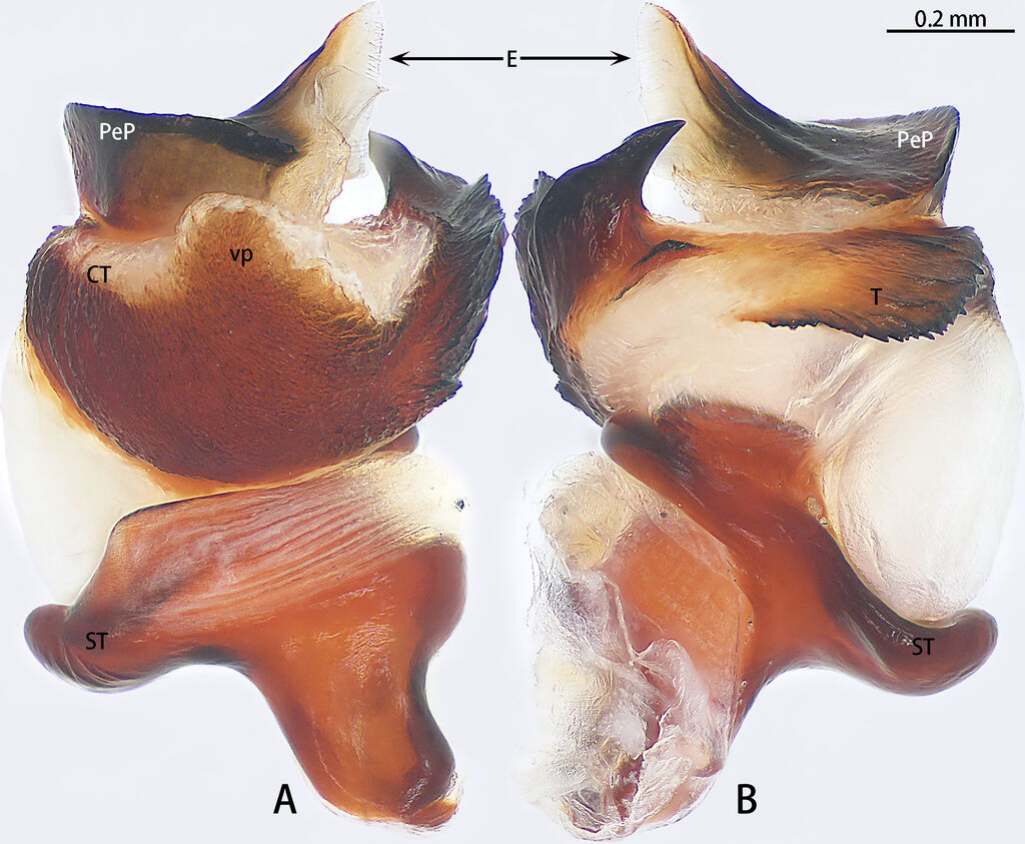
Dissected bulb of *Liphistiusliz* sp. nov., holotype male. **A** prodorsal view; **B** ventral view. Abbreviations: **CT**, contrategulum; **E**, embolus; **PeP**, paraembolic plate; **ST** subtegulum; **T**, tegulum, **vp**, ventral process of contrategulum.

**Figure 4.**
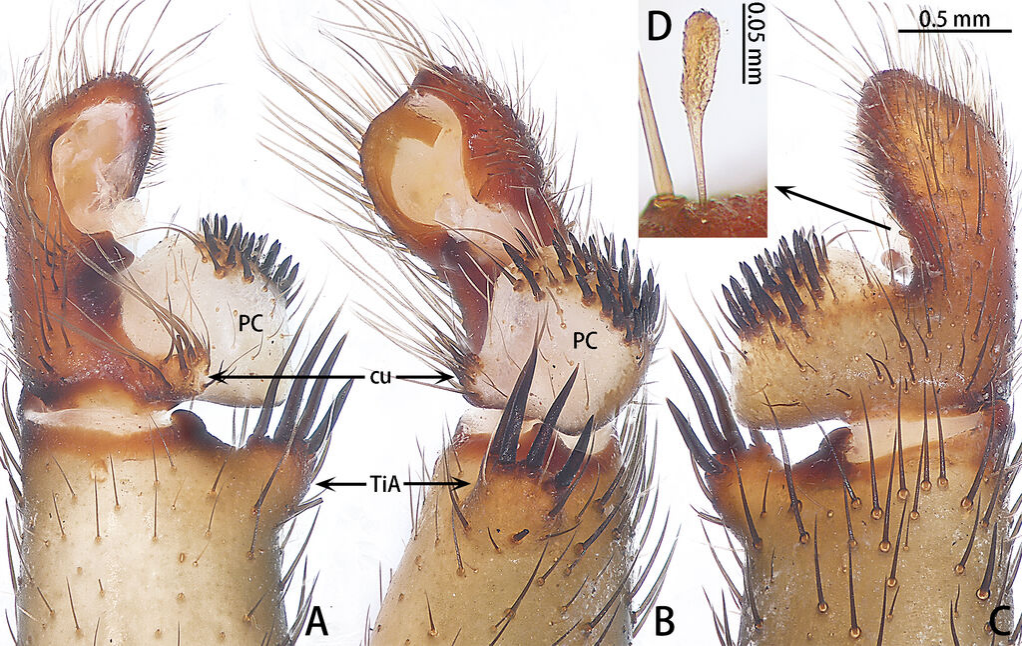
Palp of *Liphistiusliz* sp. nov., holotype male. **A** dorsal view; **B** retrolateral view; **C** ventral view; **D** clavate trichobothria. Abbreviations: **cu**, cumulus; **PC**, paracymbium; **TiA**, tibial apophysis.

**Figure 5.**
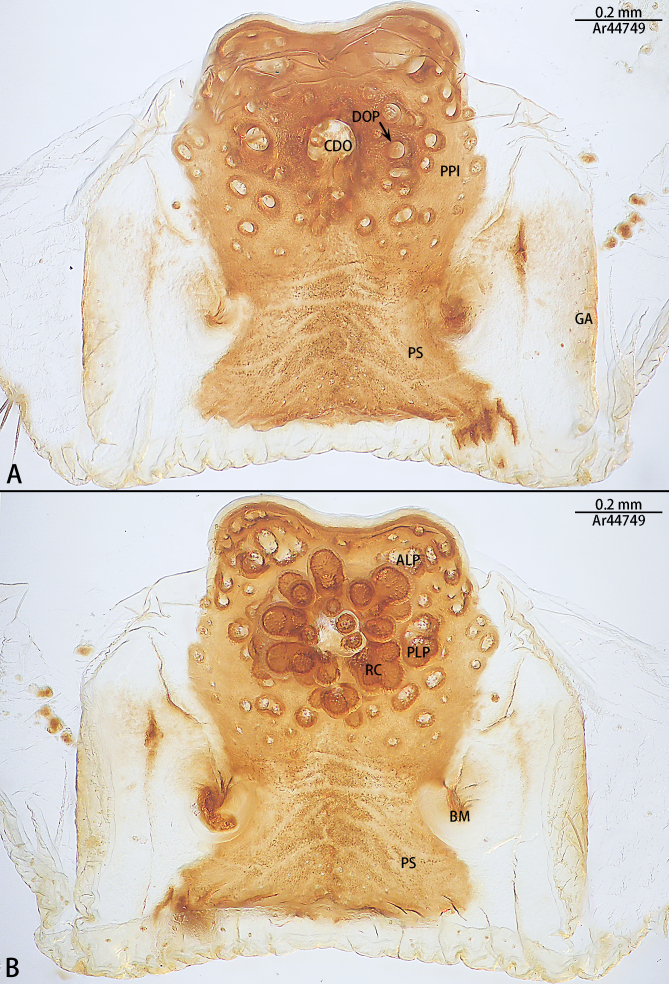
Vulva of *Liphistiusliz* sp. nov., paratype female. **A** dorsal view; **B** ventral view. Abbreviations: **ALP**, anterolateral protuberance on poreplate; **BM**, bulging margin on ventral poreplate; **CDO**, central dorsal opening; **DOP**, dorsal opening of posterolateral protuberance on poreplate; **GA**, genital atrium; **PLP**, posterolateral protuberance on poreplate; **PPl**, poreplate; **PS**, posterior stalk; **RC**, receptacular cluster.

**Figure 6.**
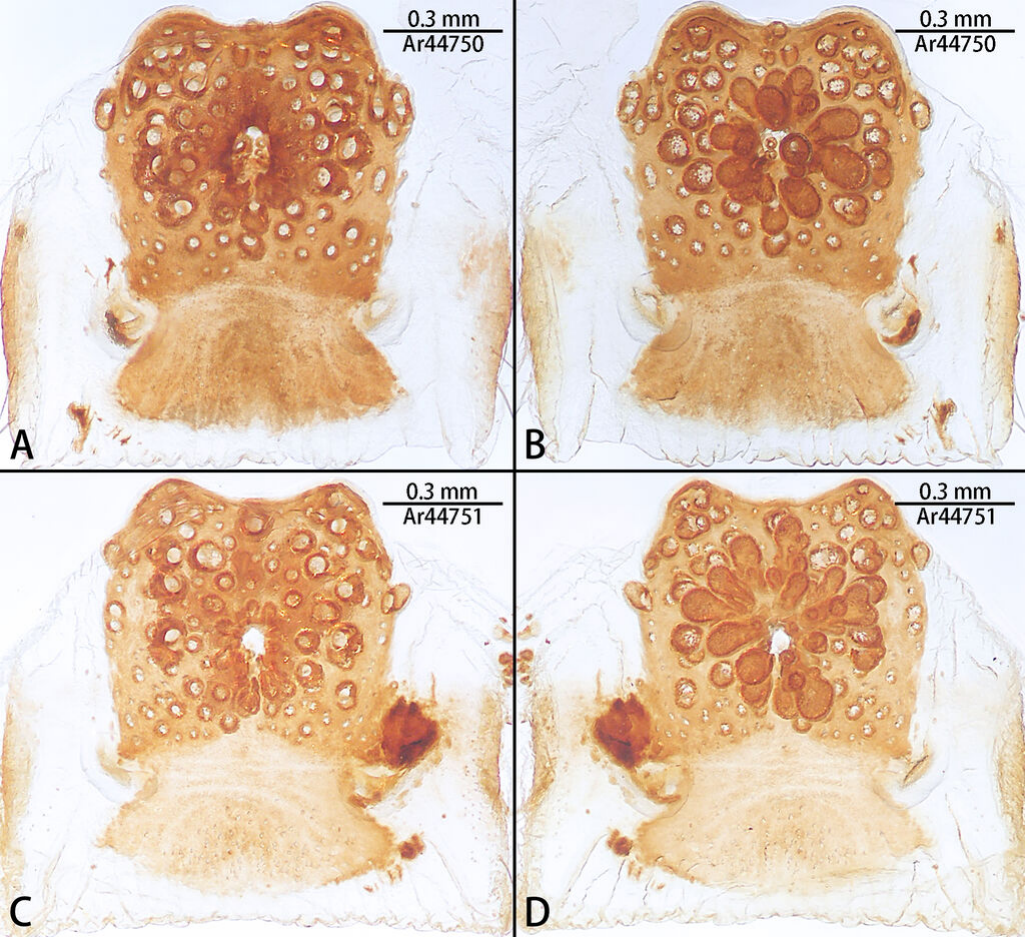
Vulvae of *Liphistiusliz* sp. nov., paratype females. **A, C** dorsal view; **B, D** ventral view.

**Figure 7.**
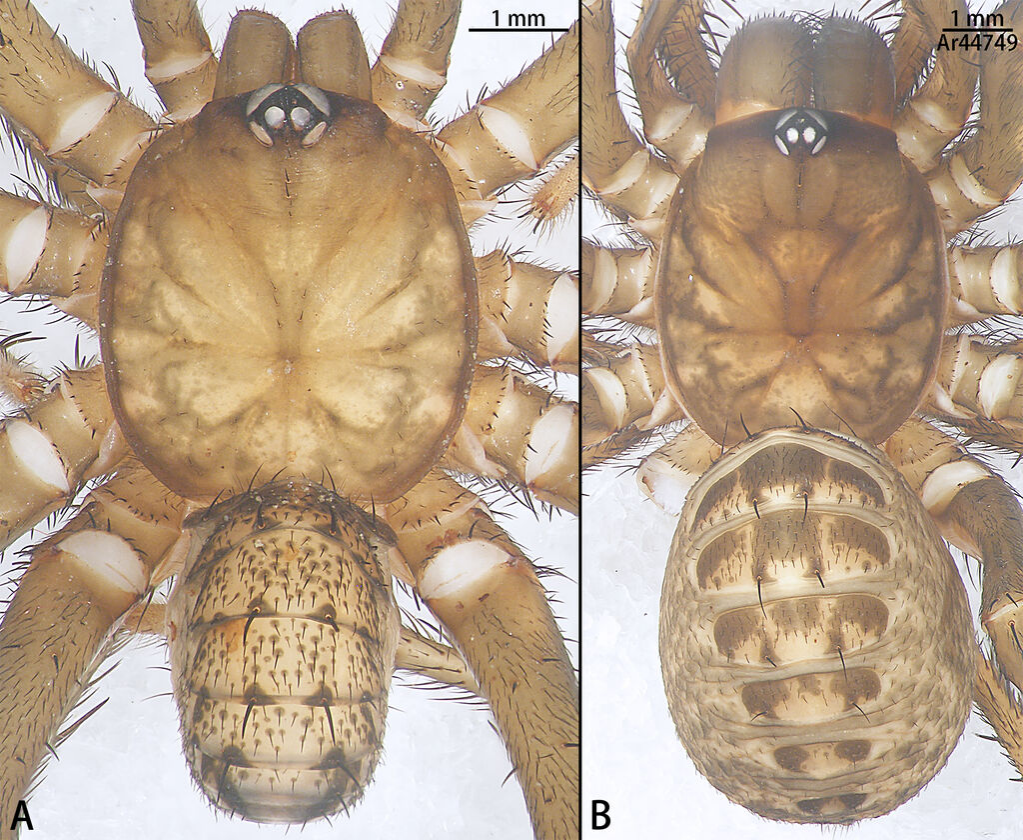
Habitus of *Liphistiusliz* sp. nov., dorsal view. **A** holotype male; **B** paratype female.

Males of the new species resemble *Liphistiusnabang* Yu, Zhang & Zhang, 2021 by the general shape of the embolus and tegulum with a clearly outlined distal edge (Fig. 3) and similar body colouration (Fig. 7) and the female with a similar-shaped poreplate plate. However, *L.liz* sp. nov. can be distinguished by the male with curved subtegulum (Fig. 2) [vs. subtegulum straight in *L.nabang* (see Yu et al. (2021), figs. 3A and B)] and tibial apophysis almost as high as wide (Fig. 4) [vs. wider than high in *L.nabang* (see Yu et al. (2021), figs. 3 D–F)]. Females of the new species can be distinguished from those of *L.nabang* by the straight genital atrium (Figs 5, 6) [vs. genital atrium curved in *L.nabang* (see Yu et al. (2021), fig. 4)], posterior stalk and poreplate are located in the same plane (Figs 5, 6) [vs. posterior stalk perpendicular to poreplate in *L.nabang* (see Yu et al. (2021), fig. 4)] and posterior stalk two times longer than wide [vs. posterior stalk four times longer than wide in *L.nabang* (see Yu et al. (2021), fig. 4)].

#### Etymology

The specific name refers to the short name for the Laboratory of Invertebrate Zoology (LIZ), Institute of Zoology, Chinese Academy of Sciences in Beijing; noun in apposition. LIZ was founded by Shen Jia-Rui (see Dai (1997)) in 1928, later led by Daxiang Song (see Marusik (2008)) from 1975 to 1995 and has been led by the senior author Shuqiang Li from 1995 to the present.

#### Distribution

**Figure 8.**
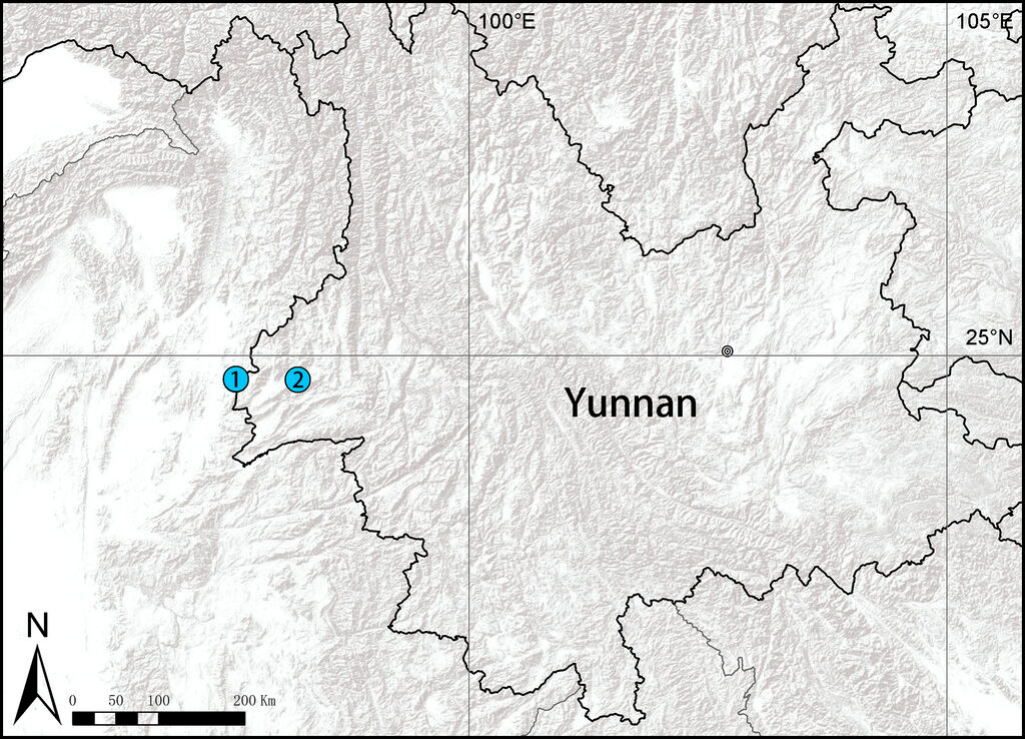
Distribution records of *Liphistius* from China. **1** *L.nabang*; **2** *L.liz* sp. nov.

China (Yunnan; Fig. 8).

#### DNA barcode

CTGCGATGGTTATATTCAACAAATCACAAAGATATTGGAACTATATATTTAATTTTTGGTGTATGATCTGCCATAATCGGAACTGCACTAAGATTATTAATTCGAGCAGAATTAGGTCAACCAGGAAGATTAATCGGAGACGATCAAACATATAATGTAATTGTAACAGCTCATGCTTTTATTATAATTTTTTTTATAGTTATACCTATAATAATTGGAGGTTTTGGAAATTGATTAATCCCTCTTATACTAAGAGCCCCTGATATAGCTTTTCCTCGATTAAATAATTTAAGATTTTGATTATTACCCCCCTCTATCACCCTCTTATTGATTTCATCCATAGTAGAAAGAGGCTCCGGCACAGGTTGGACTATTTATCCCCCTATTGCTAGCATAGAATTTCACCCTGGTATATCTATTGATTATACTATTTTTTCATTACACCTTGCCGGGGCCTCTTCAATCTTAGGCGCAATTAATTTTATTACCACTATTATTAACATACGACCAAGAGGTATATTAATAGAGCGAGTACCATTATTTGTTTGATCTATTCTTATTACCGCAAGCCTACTGTTACTATCTTTACCTGTATTAGCTGGTGCGATTACTATGCTATTAACAGATCGAAATTTTAACACGTCATTTTTTGATCCAGCAGGAGGTGGTGACCCTATCCTATTCCAACATTTATTTTGATTTTTTGGTCATCCAGAAGTTTACATTCTTATTATTCCAGGTTTTGGGATAATTTCACATATTGTAAGACACAACGCTGGAAAAAAAGAACCTTTTGGGTCTTTAGGCATAATTTATGCAATATCCGCTATTGGATTACTAGGGTTTGTAGTCTGAGCACACCATATATTTACAGTAGGTATAGATGTTGATACACGAGCTTATTTCACAGCAGCAACCATAATTATTGCAATCCCCACAGGAATTAAAATTTTTAGATGATTAGCTACTCTTCATGGTACTAATTTAATCATAAGTACTTCCCTAATATGGTCTATTGGATTTATCTTCCTATTCACTATTGGTGGATTAACAGGCGTAATCCTAGCTAATTCATCTATTGATATTGTTCTTCATGATACATACTATGTAGTAGCTCATTTTCATTATGTTTTATCAATAGGAGCAGTTTTTGCAATTATAGCAAGAATTATTCACTGATTCCCTTTATTTTTTGGATTTTCATTTAATCAAACTTTATTAAAAATTAACTTTTTTTCCATATTTATTGGTGTAAATATAACCTTTTTCCCACAACACTTCTTAGGATTAAATGGAATACCACGACGATATTCAGATTACCCTGATATATTTATATCATGAAATGTAATTTCATCTTTAGGAAGAATTTTATCTTTTCTAGCAGTAATTATATTTATTTTAATTGTATGAGAAAGAATTATATCGAACCGTAATATTTATATTCCTACTCAATCACCTTCTTCAGTTGAATGAACTCAAAATATTCCTCCTTCTAATCATACCTTTAATCAACTCAATATACTCATTTTCTAA (GenBank accession number OR721885).

#### Compared material examined

*Liphistiusnabang*: Holotype: ♂ (MHBU-ARA-00020000), CHINA, Yunnan Province, Dehong Dai and Jingpo Autonomous Prefecture, Yingjiang County, Nabang Town, 24.7521°N, 97.563°E, 265 m elev., 2 August 2019, leg. Quanyu Ji.

#### Variation

Vulvae of two paratype females, see Fig. 6.

## Supplementary Material

XML Treatment for Liphistius liz

## Figures and Tables

**Figure 3a.**
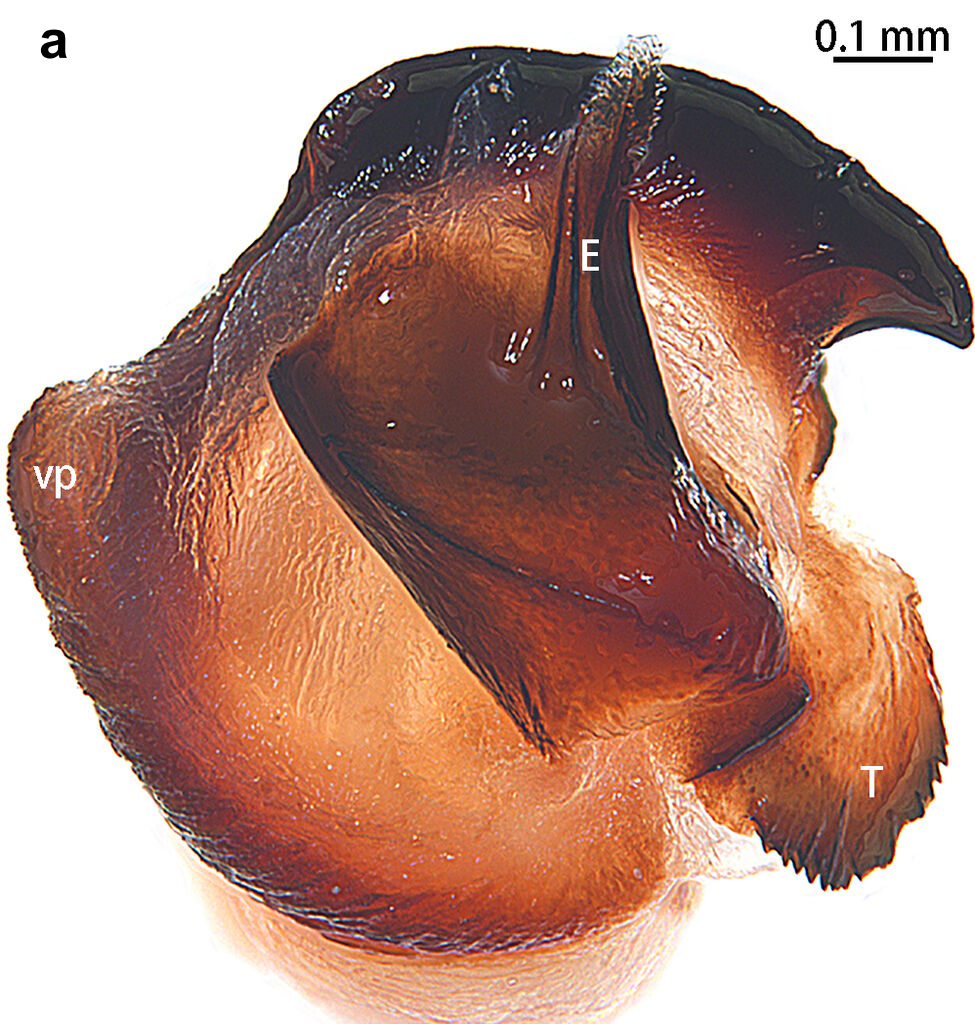
*Liphistiusnabang*, holotype male;

**Figure 3b.**
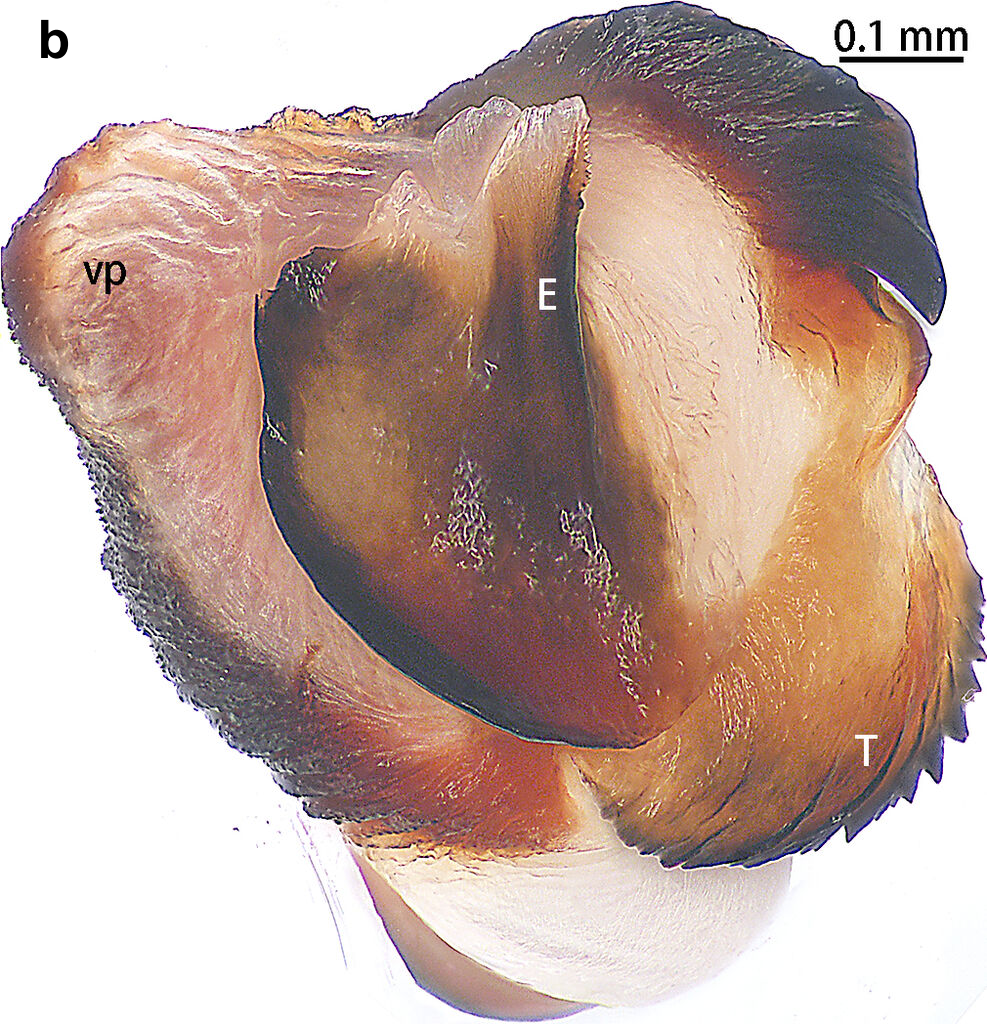
*Liphistiusliz* sp. nov., holotype male.
